# Gender Differences in Depression Scores of Iranian and German Medical Students

**Published:** 2014

**Authors:** Jamshid Ahmadi, Nahid Ahmadi, Fereshteh Soltani, Fatemeh Bayat

**Affiliations:** 1 Professor and Founder Director, Substance Abuse Research Center; Dual Diagnosis Ward; Shiraz University of Medical Sciences; Shiraz; Iran.; 2 General Physician, Substance Abuse Research Center, Shiraz University of Medical Sciences, Shiraz, Iran.; 3 Pharmacist, School of Pharmacy, Düsseldorf University, Germany.

**Keywords:** Depression, Gender Differences, German, Iranian, Medical Students

## Abstract

**Objective:**

The aim was to evaluate gender differences in depression scores of Iranian and German medical students.

**Methods:**

Two hundred Iranian medical students (100 men and 100 women) and 200 German medical students (100 men and 100 women) were selected randomly and completed the English form of the self-rating Beck Depression Inventory (BDI).

**Results:**

Analysis gave a mean rating of 10.7 ± 6.6 for Iranian men and 10.9 ± 7.81 for Iranian women (NS). Also, 5 ± 4.9 for German men and 5.6 ± 5.0 for German women (NS). On Item 2, which asked whether the person was pessimistic 33% of Iranian men and 30% of Iranian women indicated that they were pessimistic (NS). Also, 21% of German men and 20% of German women indicated that they were pessimistic (NS). On Item 9, which asked about suicidal tendencies, 9% of Iranian men and 13% of Iranian women reported as having suicidal tendencies (NS). Also, 13% of German men and 21% of German women reported as having self-harming thoughts (NS).

**Conclusion:**

The present study showed no gender differences in Iranian and German medical students’ scores on the BDI.

## Introduction

There is sufficient evidence in literature that the adaptive capacities of medical students are decreased by psychiatric symptoms (1-3). Depression, dysthymia, and dysphoric mood have also been associated with medical school dropout (2). Healthy medical students are likely to become healthy physicians who can then model and promote healthy lifestyles with their patients. Depression can decrease the functionality of people, especially the medical students and disturb the relationship among the medical students and also between physician and patient (3, 4). A significant number of surveys about mental health of men and women indicated that women are more prone to depression than men (2, 5). On the other hand, a number of studies have shown that there was no sex difference on depression (6, 7). There is some evidence that psychiatric disorders especially depression during medical school predicts later job problems in physicians (8). It is mentioned that physicians cannot provide the kind of special help for themselves as they would for their patients (9-11). It appears that medical students follow a similar behavior (12, 13). However, little is known about the prevalence of psychological disturbances, especially depression. Based on a research study, using the General Health Questionnaire-12, 22-36% of medical students complained of having emotional disorders (14). Medical students reported more depression than the general population even before entering medical school (15). According to Beck Depression Inventory (BDI) scores, prevalence rates of depression have been mentioned to be in the range of 14-24% (3, 16, 17). One hundred and forty Hong Kong Chinese students were assessed early in their 2^nd^ year of medical school and were compared with 138 students evaluated at the beginning of their 1^st^ year of medical school and with 74 non-medical university students in their 2^nd^ year. Based on their self-reported scale scores, anxiety, and depression were higher in 2^nd^ year medical students (18).

It has been revealed and well-documented that healthy medical students are most likely to become healthy physicians who can then model and promote healthy lifestyles with their patients. Dysphoric mood and depression decreases the achievement of medical students and disturbs their relationships with patients (2, 3, 19-23).

According to a study undertaken at Shiraz University of Medical Sciences in Iran, using BDI, the mean scores of depression among 651 students were: 12 for physiotherapy students, 11.2 for junior medical students, 9.7 for senior medical students, and 6.2 for resident physicians (19). In this study, female respondents were reported to have more depression than male respondents. In another survey at Shiraz University in Iran, the mean BDI scores among Iranian non-medical students were 11.6 for men and 9.9 for women (20).

Since medical students most directly and frequently interact with patients, they are likely to affect patients by their verbal and non-verbal behavior. Therefore, it is of interest to explore depression, dysphoric mood, hopelessness, and suicidal thoughts among them. The main objective of this research study was to assess the rate and gender differences in depression among Iranian and German medical students who are representatives of two different cultures.

## Materials and Methods

***Subjects***

The subjects were 200 Iranian medical students (100 men and 100 women) of Shiraz University of Medical Sciences (in Asia continent) and 200 German medical students (100 men and 100 women) of Dusseldorf University (in Europe continent), selected by assigned cluster random sampling from clinical medical students in Iran as the representative of Eastern Mediterranean Region and also Germany as the representative of Europe. The clusters cover different grades of medical students.

The inclusion criteria were: 1) being a medical student at Shiraz University of Medical Sciences, or at Dusseldorf University, 2) having Iranian or German nationality.

***Instrument***

The English form of the BDI (21-item version) was used both for the Iranian and the German medical students. 

The BDI, a culture free set of 21 items, requires self-report by subject (24-26). According to Hersen and Bellack (1985): “Clinical Behavior Therapy with Adults”, New York, Plenum Press, the minimum score is 0 and the maximum is 63. Ratings from 0 to 9 are considered in the normal range, 10 to 19 marginal, 20 to 29 moderate, 30 to 39 moderate to severe, and 40 or above severe depression (26).

In addition to a total score on all items, Item 2 (I am not particularly pessimistic/I am pessimistic or discouraged about the future) and Item 9 (I do not have any thoughts of harming myself/I have thoughts of harming myself) were analyzed separately for pessimism and self-harming tendencies among students.

The questionnaires were distributed among the students and were followed by a full explanation of the reasons for conducting the study. The students were informed about the confidentiality of their responses. In addition, special attention was paid to ensure that they clearly understand the instructions about answering the questionnaire. In order to encourage them to provide honest and more open answers, the students were asked not to write their names or student card numbers on questionnaires. The students were given enough time to answer and return the BDI. All students completed the questionnaire. The study was approved by both of the university authorities.

***Analysis***

Data analysis was performed with SPSS program (version 16.0; SPSS Inc., Chicago, IL, USA). A chi-square test was used to test the frequencies, t-test was used to compare the means (the mean score on the BDI) and p = 0.05. P-values ≤ 0.05 are reported as “statistically significant” and P-values more than 0.05 are reported as “statistically non-significant (NS)”.

## Results

The mean score on the BDI of Iranian medical students was 10.76 ± 7.23 and for German medical students was 5.36 ± 4.91 (p < 0.05). The mean score on the BDI was 10.65 ± 6.63 for Iranian men and 10.87 ± 7.81 for Iranian women (NS), which is consistent with a prior report in Iran (27). Also, 5.11 ± 4.88 for German men and 5.61 ± 4.95 for German women (NS), which shows no significant gender difference. On Item 2, which asked whether the person was pessimistic 33% of Iranian men and 30% of Iranian women indicated that they were pessimistic (NS). Also, 21% of German men and 20% of German women indicated that they were pessimistic (NS). On Item 9, which asked about suicide, 9% of Iranian men and 13% of Iranian women scored as having suicidal thoughts (NS), while 13% of German men and 21% of German women scored as having suicidal thoughts (NS). The findings of the present study showed no gender differences among the Iranian and German medical students. It was also concluded that more depression was reported among Iranian medical students than among the Germans.

## Discussion

To the best of our knowledge, there has not been any paper published yet to compare depression of Iranian and German medical students. More research needs to be done in different cultures to find gender differences on depression.

As in all epidemiological and self-report surveys our research study has a couple of limitations including recall bias (considering that the BDI measures the current depression); therefore, it represents a major threat to the internal validity of our study using self-reported data. Tendency of medical students to report past events in a manner that is different from reality and reporting errors should be considered carefully. In spite of assuring the confidentiality, there is a probability that some of the students did not answer all the questions accurately; therefore, there is a probability of underreporting. 

## Notes

**Declaration of interest:** None.

## References

[1] Reuben DB (1985). Depressive symptoms in medical house officers Effects of level of training and work rotation. Arch Intern Med.

[2] Clark DC, Zeldow PB (1988). Vicissitudes of depressed mood during four years of medical school. JAMA.

[3] Ahmadi J (1991). [Behavior therapy].

[4] Ahmadi J (1992). [Future of psychiatry].

[5] Weissman MM, Klerman GL (1977). Sex differences and the epidemiology of depression. Arch Gen Psychiatry.

[6] Leighton AH, Lambo TA, Hughes CC, Leighton DC, Murphy JM, Macklin DB (1963). Psychiatric disorder among the yorba.

[7] Bash KW, Bash-Liechti J (1974). Studies on the epidemiology of neuropsychiatric disorders among the population of the city of Shiraz, Iran. Social psychiatry.

[8] Tyssen R, Rovik JO, Vaglum P, Gronvold NT, Ekeberg O (2004). Help-seeking for mental health problems among young physicians: is it the most ill that seeks help? - A longitudinal and nationwide study. Soc Psychiatry Psychiatr Epidemiol.

[9] Toyry S, Rasanen K, Kujala S, Aarimaa M, Juntunen J, Kalimo R (2000). Self-reported health, illness, and self-care among finnish physicians: a national survey. Arch Fam Med.

[10] Rosvold EO, Bjertness E (2002). Illness behaviour among Norwegian physicians. Scand J Public Health.

[11] Tyssen R, Vaglum P, Gronvold NT, Ekeberg O (2001). Factors in medical school that predict postgraduate mental health problems in need of treatment A nationwide and longitudinal study. Med Educ.

[12] Chew-Graham CA, Rogers A, Yassin N (2003). 'I wouldn't want it on my CV or their records': medical students' experiences of help-seeking for mental health problems. Med Educ.

[13] Hooper C, Meakin R, Jones M (2005). Where students go when they are ill: how medical students access health care. Med Educ.

[14] Guthrie E, Black D, Bagalkote H, Shaw C, Campbell M, Creed F (1998). Psychological stress and burnout in medical students: a five-year prospective longitudinal study. J R Soc Med.

[15] Zoccolillo M, Murphy GE, Wetzel RD (1986). Depression among medical students. J Affect Disord.

[16] Givens JL, Tjia J (2002). Depressed medical students' use of mental health services and barriers to use. Acad Med.

[17] Tjia J, Givens JL, Shea JA (2005). Factors associated with undertreatment of medical student depression. J Am Coll Health.

[18] Stewart SM, Betson C, Marshall I, Wong CM, Lee PW, Lam TH (1995). Stress and vulnerability in medical students. Med Educ.

[19] Ahmadi J (1994). [Rate of depression among medical students, resident physicians and physiotherapy students at Shiraz University of Medical Sciences. ] Shiraz Univ J Soc Sci Hum.

[20] Ahmadi J (1994). [Rate of depression among students at Shiraz University]. J Students Res.

[21] Dahlin ME, Runeson B (2007). Burnout and psychiatric morbidity among medical students entering clinical training: a three year prospective questionnaire and interview-based study. BMC Med Educ.

[22] Ristic-Ignjatovic D, Hinic D, Jakovljevic M, Fountoulakis K, Siepera M, Rancic N (2013). A ten-year study of depressive symptoms in Serbian medical students. Acta Clin Croat.

[23] Moreira DP, Furegato AR (2013). Stress and depression among students of the last semester in two nursing courses. Rev Lat Am Enfermagem.

[24] Parteu D (1975). Research in depression. J Psychol: Iranian Psychol Associat.

[25] Beck AT, Ward CH, Mendelson M, Mock J, Erbaugh J (1961). An inventory for measuring depression. Arch Gen Psychiatry.

[26] Hersen M, Bellack AS (1985). Handbook of clinical behavior therapy with adults.

[27] Makaremi A (1992). Sex differences in depression of Iranian adolescents. Psychol Rep.

